# Postbiopsy hepatic hemorrhage due to an occult aberrant posterior sectoral hepatic artery

**DOI:** 10.1007/s00508-026-02756-2

**Published:** 2026-05-05

**Authors:** Mustafa Yeşilyurt, Fahri Aydın

**Affiliations:** Department of Radiology, Faculty of Medicine, Atatürk University, 25040 Erzurum, Turkey

A 55-year-old man with chronic kidney disease underwent ultrasound-guided percutaneous liver biopsy for evaluation of chronic parenchymal liver disease. The biopsy targeted segment VII of the right hepatic lobe using a 16-gauge automated core biopsy needle. Approximately 4 h after the procedure, the patient developed a marked decrease in hemoglobin level from 8.8 g/dL to 6.3 g/dL, although he remained hemodynamically stable. Contrast-enhanced computed tomography demonstrated a subcapsular hematoma with a suspected bleeding focus along the posterior aspect of the right hepatic lobe (Fig. 1a). In addition, three-dimensional reconstruction demonstrated an aberrant posterior sectoral hepatic artery arising directly from the abdominal aorta, independent of the celiac trunk (Fig. 1b). The patient was referred for angiographic evaluation. Selective catheterization of the celiac trunk and common hepatic artery demonstrated conventional hepatic arterial anatomy without evidence of contrast extravasation; however, the posterior segments of the right hepatic lobe appeared relatively poorly opacified compared with the anterior segments, suggesting an alternative arterial supply (Fig. 2a). Given the discrepancy between the clinical findings and the initial angiographic results, abdominal aortography was performed. This demonstrated an aberrant arterial branch arising directly from the abdominal aorta and supplying segments VI–VII of the right hepatic lobe (Fig. 2b). Superselective catheterization of this artery revealed active contrast extravasation at the level of segment VII, corresponding to the biopsy site (Fig. 2c). Transarterial embolization with detachable coils was successfully performed. Postembolization angiography demonstrated complete occlusion of the bleeding vessel with no residual hemorrhage (Fig. 2d). The patient remained clinically stable and no further decline in hemoglobin level was observed.

**Fig. 1:**
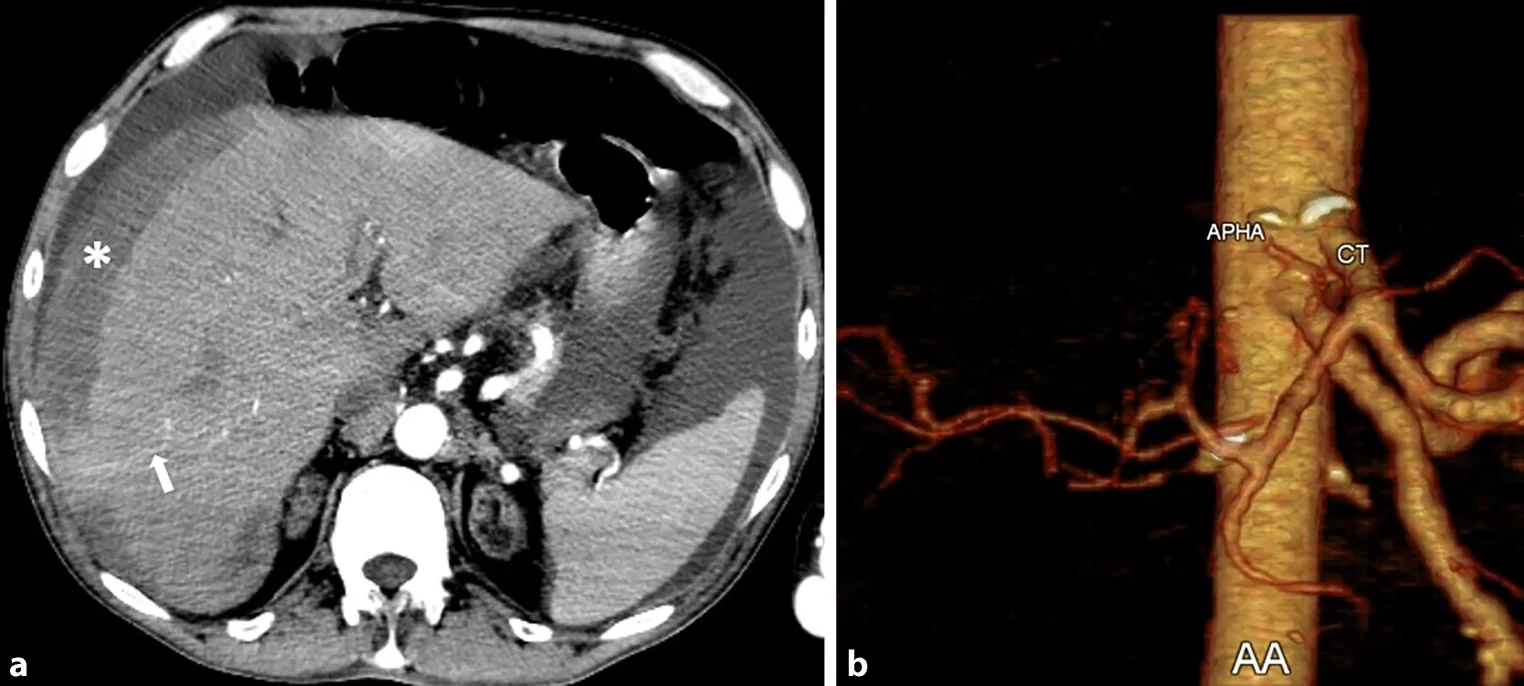
**a** Contrast-enhanced computed tomography demonstrating a subcapsular hematoma (asterisk) with a suspected bleeding focus (arrow) in the posterior right hepatic lobe. **b** Three-dimensional reconstruction demonstrating an aberrant posterior sectoral hepatic artery (*APHA*) arising directly from the abdominal aorta (*AA*), independent of the celiac trunk (*CT*)

**Fig. 2:**
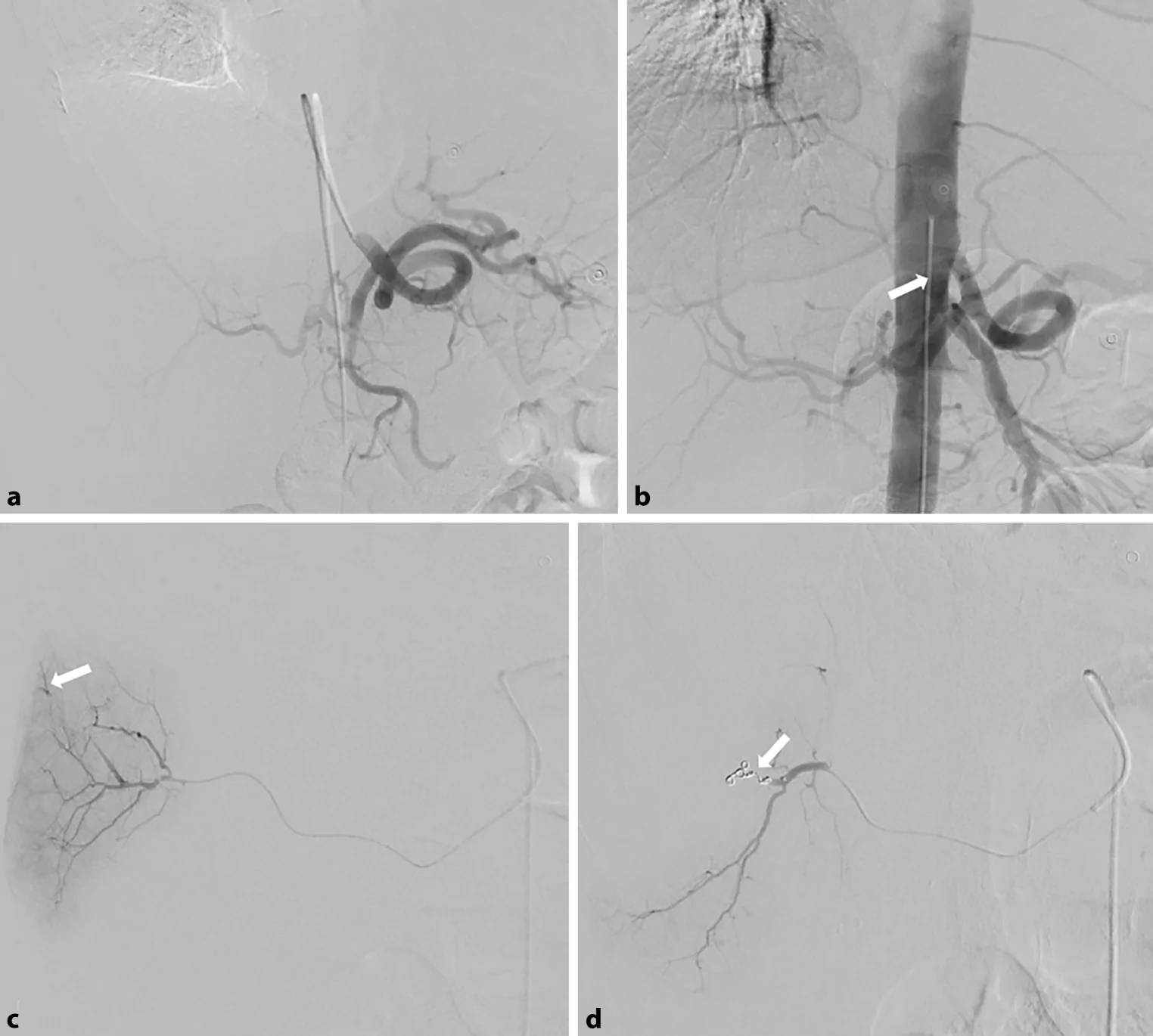
Angiographic findings and treatment: **a** Selective celiac angiography showing no contrast extravasation. **b** Abdominal aortography demonstrating an aberrant artery arising directly from the abdominal aorta (arrow). **c** Superselective angiography revealing active contrast extravasation in segment VII (arrow). **d** Postembolization angiography showing complete occlusion with no residual bleeding and embolization coils within the treated branch (arrow)

Hemorrhage is the most significant complication of percutaneous liver biopsy [1, 2]. Although selective celiac angiography is usually sufficient to identify the bleeding source, variant arterial anatomy may lead to false negative findings. In the present case, the source of bleeding was an occult aberrant posterior sectoral hepatic artery arising directly from the abdominal aorta and supplying segments VI–VII of the right hepatic lobe. The hepatic arterial system demonstrates considerable anatomical variability, most commonly involving replaced or accessory arteries arising from the superior mesenteric or left gastric arteries; however, direct arterial supply from the abdominal aorta is exceedingly rare [3, 4]. In patients with persistent suspicion of postbiopsy hemorrhage despite negative initial angiography, aortography may be essential for identifying occult bleeding sources and guiding appropriate endovascular treatment [2–4].

## Data Availability

The data supporting the findings of this study are not publicly available due to patient privacy and ethical considerations but are available from the corresponding author upon reasonable request.
